# Sodium hyaluronate as a drug-release system for VEGF 165 improves graft revascularization in anterior cruciate ligament reconstruction in a rabbit model

**DOI:** 10.3892/etm.2012.629

**Published:** 2012-07-03

**Authors:** JIARONG CHEN, LIU YANG, LIN GUO, XIAOJUN DUAN

**Affiliations:** The Center for Joint Surgery, Southwest Hospital, The Third Military Medical University, Chongqing 400038, P.R. China

**Keywords:** sodium hyaluronate, vascular endothelial growth factor, allograft, revascularization, anterior cruciate ligament

## Abstract

Graft remodeling following anterior cruciate ligament (ACL) reconstruction requires a long period of recovery in which vascular endothelial growth factor (VEGF) plays an important role. The short half-life of exogenous VEGF, however, restricts its use. The aim of this study was to investigate sodium hyaluronate (SH) as a delivery system for VEGF in graft revascularization. Non-cumulative release into phosphate-buffered saline (PBS) was firstly measured spectrophotometrically for 1–4 days. Allogeneic bone-patellar tendon-bone (B-PT-B) was soaked in the VEGF/SH formulation and implanted in a rabbit model to regenerate the ACL and observe the vascularization and biomechanical properties. The results revealed that a steady state was achieved after ∼40 h in non-cumulative measurements. The release plotted as a function of the square root of time was consistent with a largely diffusion-controlled release system. At 2, 4 and 8 weeks, the microvessel density of grafts was higher in the VEGF/SH-treated group compared to the control groups. Although there was a temporary decline at 2 weeks, the stiffness and maximum tensile load of the experimental group was significantly greater than that of the control group at 4 and 8 weeks (P<0.01). Our findings suggest that SH can be used as a good carrier of VEGF, which can improve the early revascularization and biomechanical properties of B-PT-B allografts after ACL reconstruction.

## Introduction

The anterior cruciate ligament (ACL) plays an important role in the stability of the knee joint. If it fails to heal after complete rupture, premature osteoarthritis and disability can result (1). At present, ACL reconstruction is considered as the standard treatment following injury. Allogeneic bone-patellar tendon-bone (B-PT-B) for ACL reconstruction is one of the most important ligaments (2). Furthermore, it is widely known that the healing process of the grafted tendon involves four main stages: necrosis, angiogenesis, cell repopulation and final maturation, among which, angiogenesis is an essential step in the process of tendon healing and graft remodeling in which revascularization prompts delivery of inflammatory cells, fibroblasts and growth factors to the wound site (3). A clinical case report suggests that the tendon grafts do not recover to physiological levels even 18 months after surgery (4).

Therefore, we tried to develop a new strategy by enhancing angiogenesis to accelerate the remodeling of tendon graft. Vascular endothelial growth factor (VEGF) plays an essential role in angiogenesis, regulating the activation, migration and proliferation of endothelial cells in various pathological conditions (5). VEGF is easily decomposed *in vivo*, however, which makes it easy to lose its biological effects. If there was a method to make VEGF release slowly, consequently, it would be able to promote revascularization and improve the quality of graft survival.

Sodium hyaluronate (SH), a derivative of hyaluronate acid (HA), was discovered in bovine vitreous humour by Meyer and Palmer in 1934 (6). It is well-tolerated, safe and efficacious and has been commonly used as a growth scaffold in surgery, wound healing and embryology (7). In addition, administration of purified high-molecular-weight SH into orthopedic joints can restore the desirable rheological properties and alleviate some of the symptoms of osteoarthritis. Previous studies have shown that SH could be used as a carrier of drugs (8), delaying the drug release rate.

Based on these two points, we hypothesized that an application of VEGF mixed with SH enhances angiogenesis of a graft by prolonging the action time of VEGF in ACL reconstruction, and the application does not affect the mechanical characteristics of the ACL graft. The aim of this study was to test these hypotheses by a rabbit ACL reconstruction model using the B-PT-B graft.

## Materials and methods

### Experimental design

VEGF 165, 1 and 2.3% SH were mixed to yield SH formulations containing VEGF 165 in concentrations of 5–10 μg/ml. Non-cumulative release into phosphate-buffered saline (PBS) was first measured spectrophotometrically over 1–4 days to detect the release kinetics of VEGF. Allogeneic B-PT-B was then soaked in the VEGF/SH formulations and implanted in the rabbit model to regenerate ACL. Briefly, 45 skeletally mature female New Zealand rabbits (90 limbs) weighing 3.0–3.5 kg supplied by the Animal Centre of the Third Military Medical University (Chongqing, China) were then randomly divided into 5 groups (groups A–E). From groups A–D, ACL was transected at each hind limb, and then replaced by allogeneic B-PT-B. In group A (n=9), B-PT-B allografts soaked in VEGF 165 and SH were transplanted into the knee joints. In group B (n=9), B-PT-B allografts soaked in VEGF 165 were transplanted. In group C (n=9), B-PT-B allografts soaked in SH were transplanted. In group D (n=9), B-PT-B allografts soaked in PBS were transplanted. In group E (n=9), no treatment was applied but an incision of the capsule was performed. Six limbs were harvested from each group at 2, 4 and 8 weeks after surgery for biomechanical analysis, 3 of which were then used for immunohistological evaluations for VEGF, and the other 3 for CD31, which is a marker for vascular endothelial cells. All studies involving animals were approved by the Institute’s Animal Care and Use Committee.

### Release kinetics of VEGF

VEGF (VEGF 165; Peprotech, Inc., Rocky Hill, NJ, USA) at concentrations of 5 or 10 μg/ml, respectively, and 1 or 2.3% SH (Sigma, USA), respectively, were mixed. A total of 0.2 ml of the gel was placed into 0.2 ml of receiver fluid of PBS. The samples were incubated at 37°C. Release kinetics were measured as non-cumulative release (8), with measurements at 3, 6, 12, 18, 24, 48 h and 4 days. Because the receiver fluid was not replaced, each container was measured only once. For every single concentration and time point at least eight samples were examined. VEGF 165 concentration in the supernatants was assayed spectrophotometrically at 239 nm (Ultrospec 1000; Pharmacia Biotech). To determine whether the system of SH/VEGF represents a diffusion-controlled or a membrane-limited release system, the initial release was examined carefully by preparing a large amount of samples as described above and taking measurements at 5 min intervals (each single sample was measured only once). The release of VEGF was plotted as a function of the square root of time.

### Graft preservation

A total of 45 B-PT-B were harvested from donor New Zealand white rabbits (from other experiments). Each graft was separated into at least two bundles of ligament and each bone was cut 4-mm wide and 10-mm long. All the grafts were enclosed in tubes and placed in dry ice in a container for γ irradiation by cobalt-60 (Co-60) for 9 h yielded at a dose of 2.5 Mrad. The tendon grafts were then stored at −80°C for >3 months for later use.

### Preoperative preparation of B-PT-B allografts

Thirty minutes before surgery, the graft for group A was soaked in 1% SH formulations containing VEGF 165 at a concentration of 5 μg/ml as described previously (9) with 10 ml PBS for 30 min; the graft for group B was soaked in recombinant human VEGF (5 μg/ml) with 10 ml PBS for 30 min; the graft for group C was soaked in SH (100 μg/ml) with 10 ml PBS for 30 min. In group D, the graft was soaked in 10 ml PBS for 30 min.

### Surgical procedure

**Figure 1:**
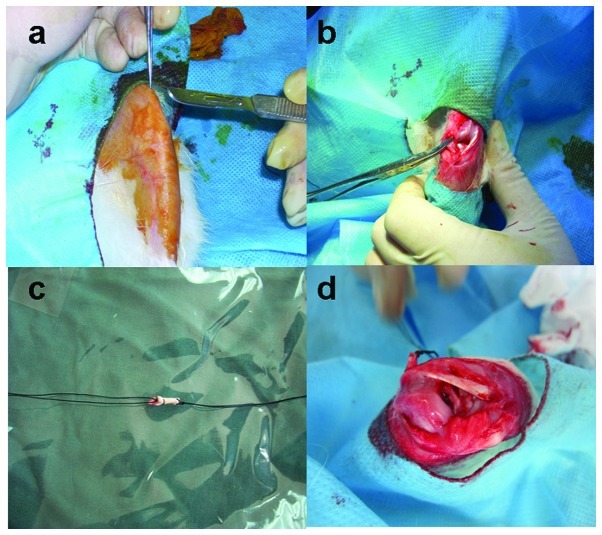
The surgical procedure for ACL reconstruction using B-PT-B allograft. (a) Medial parapatellar incision was performed. (b) The ACL was transected with a retrograde knife at its femoral attachment under visual control. (c) B-PT-B allograft was pretreated with different solution before transplantation. (d) The allograft was inserted via the holding suture from the tibial and femoral tunnels and then sewed to a screw inserted into the bone.

The experimental animal was anesthetized with intravenous pentobarbital sodium (0.03 g/kg). Using a sterile technique, the ACL was exposed through a medial parapatellar incision (Fig. 1a) and transected with a retrograde knife at its femoral attachment under visual control (Fig. 1b). The bone tunnels in the femur and tibia were made at the centers of the insertion sites of the ACL. The tibial tunnel was reamed to 2 mm over a guide pin. For the femoral tunnel, a 2-mm cannulated drill bit was inserted over a guide pin from the inside of the knee joint. The graft (Fig. 1c) was then inserted via the holding suture from the tibial and femoral tunnels at the same time slowly. Each end of the graft was sewed to a screw inserted into the bone. Then the incision was routinely closed in layers (Fig. 1d). After surgery, all the animals were allowed to move freely in their cages.

### Biomechanical research

At 2, 4 and 8 weeks following surgery, 6 limbs were harvested from each group. The hind limbs were removed at the hip and cut 3 cm above and below the knee joint line. Next, femur-graft-tibia specimens were prepared, removing all periarticular and intra-articular soft tissues, respectively. RGT-5KN microcomputer control electron omnipotent biomechanical testing machine (Model RGT-5KN; Shenzhen Shenke Medical Instrument Technical Development, Co., Ltd., China) was used for biomechanical testing of the graft and the normal ligament at each time point. The femur-graft-tibia complex was placed vertically between two clamps of the tensiometer. When the tension reached 2.0 N/m, we measured the length of the graft 3 times, taking its average as ligament length. Before the tensile test, the specimen was preconditioned with a static preload of 5.0 N for 10 min, followed by 10 cycles of loading and unloading with a strain of 0.5% at the crosshead speed of 50 mm/min. Subsequently, the complex underwent tensile testing at the crosshead speed of 50 mm/min until the complex failed. Applied maximum load was recognized as the maximum force and stress withheld before wound rupture.

### Histological and immunological observation

The femur-graft-tibia specimens were embedded in paraffin and cut into 5-μm thick sections longitudinal to the bony tunnels. The slides were stained with hematoxylin and eosin (H&E) and with CD31 mouse monoclonal antibody (Neomarkers, Fremont, CA, USA) to assess the endothelial cells. Three independent observers, who were blinded to the treatment groups, enumerated the microvessels of the graft under a fluorescent microscope, as described previously (10). In brief, five consecutive CD31 immunofluorescent sections were prepared for every sample. One observer selected microvessel-dense areas in every section under final magnification ×100, and randomly chose five fields under final magnification ×200. The microvessels of each randomly chosen field were counted by three independent observers, respectively. The mean of the three independent counts by the observers was considered the final counting value for each counting field. All observers followed prescheduled rules as following. Under the microscope, each luminal structure composed of endothelial cells, each separate solitary brownish-yellow endothelial cell or each cell mass within bone tissue was counted as a vessel. Vessels with a thick muscle layer or those with a luminal diameter >8 red blood cells were not counted. Areas of bleeding and fibrosis were also disregarded.

### Statistical analysis

All data were expressed as mean values ± standard deviation (SD). The statistical significance of differences in parameters was assessed by one-way analysis of variance (ANOVA) using SPSS software (SPSS v12.0; IBM, New York, NY, USA). If statistical differences between periods were found, Fisher’s PLSD tests for post hoc multiple comparisons were used. For all data collection, the investigators were blinded to the identity of the groups. The significance limit was set at P=0.05.

## Results

### Release kinetics of VEGF

**Figure 2:**
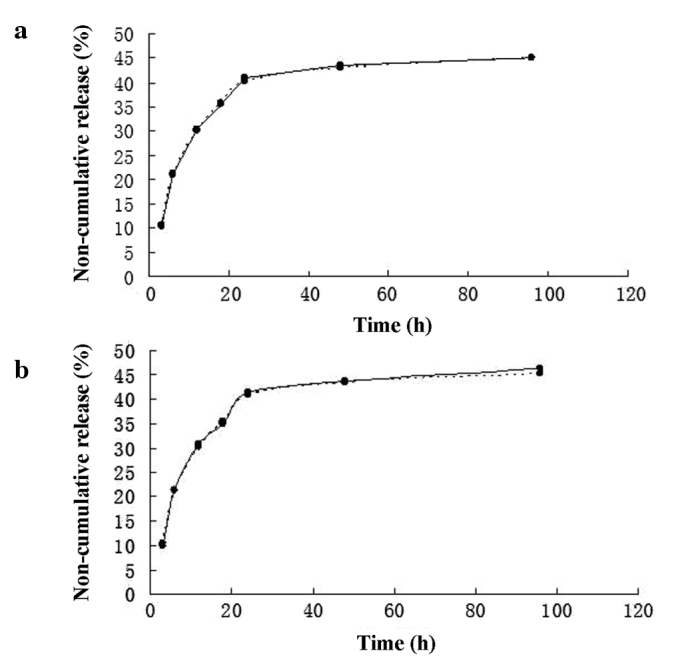
(a) Non-cumulative release of VEGF into phosphate-buffered saline (PBS) from 0.5 ml SH gel at 37°C. The loading of 1 or 2.3% SH with VEGF was 5 μg/ml (continuous curve, 1% SH; dotted curve, 2.3% SH). (b) Non-cumulative release of VEGF into PBS from 0.5 ml SH gel at 37°C. The loading of SH with VEGF was 5 (dotted curve) and 10 μg/ml (continuous curve).

The release kinetics were well-controlled and reproducible throughout the entire study. Steady state was established after 48 h. The non-cumulative release rates in 1 and 2.3% SH were basically identical (Fig. 2a). A moderate initial burst effect was noted. No kinetic differences were observed between the higher (10 μg/ml) and lower (5 μg/ml) concentration of VEGF (Fig. 2b). The kinetics of the non-cumulative release studies did not change when the volume of the receiver fluid was increased (data not shown). Finally, it was observed that 1 and 2.3% SH became less viscous and dissolved within 2 weeks.

### Biomechanical evaluation

**Figure 3:**
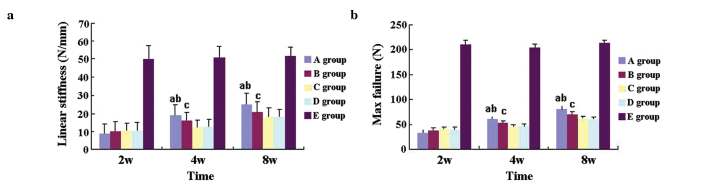
Structural properties of the femur-graft-tibia complex. (a) Linear stiffness and (b) ultimate failure load. A one-way ANOVA demonstrated significant differences in linear stiffness and ultimate failure load among group E and groups A–D, although there were no significant differences in groups C and D (P>0.05) at each analysis time. ^a^P<0.05 vs. group B; ^b^P<0.01 vs. groups C and D; ^c^P<0.05 vs. groups C and D.

All grafts failed at the middle portion during the ultimate failure testing and all normal ACL specimens had avulsion fractures at the tibial insertion sites. The linear stiffness of the FGT complex in group A was lower than that of the other groups at 2 weeks, however, the data values were increased in group A when compared with these values in the other groups at 4 and 8 weeks (Fig. 3a). The stiffness values of groups A–D were significantly lower than that of group E at every time point (Fig. 3a). The average ultimate failure of the group A was lower than the other groups at 2 weeks but it was significantly greater than that of the other control groups at 4 and 8 weeks (Fig. 3b), although there were no significant differences in the ultimate failure load between groups C and D (4 weeks, P=0.1103; 8 weeks, P=0.1302). The ultimate load values of these four groups were significantly lower than that of the normal complex (Fig. 3b).

### Histological and immunological observation

Hematoxylin staining showed that 2 weeks after surgery, in groups A and B, granulation was formed and vascularization was noted. Group B obviously had a lower cell quantity than group A. Conversely, in groups C and D most of the host cells that invaded into the graft were inflammatory cells. Four weeks postoperatively, endothelial cells gradually increased from the surface into the deep portion. Furthermore, cell volumes of the control groups were far less than groups A and B. Eight weeks postoperatively, each group revealed that endothelial cells had reached the depth of the graft.

**Figure 4:**
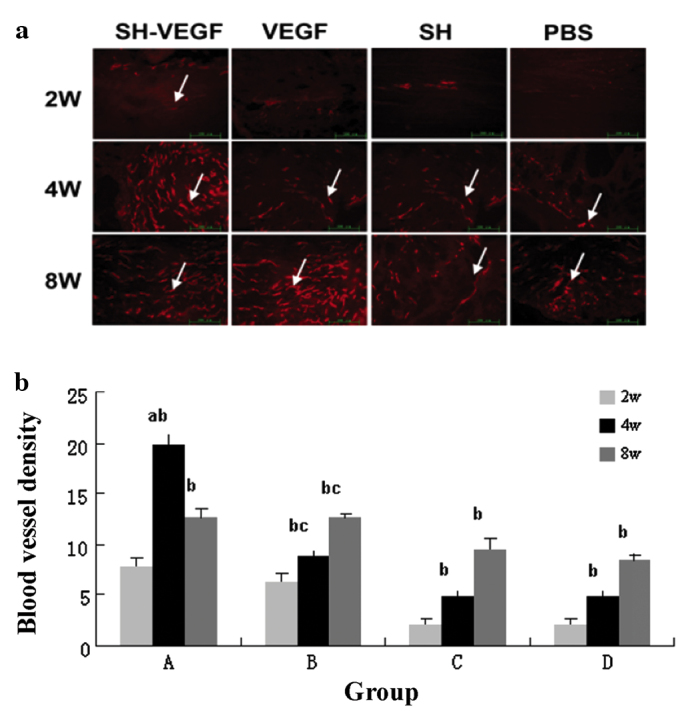
(a) Immunofluorescent staining of each group at 2 (2W), 4 (4W) and 8 weeks (8W). Magnification, ×200. The white arrow identifies the CD31-positive staining. (b) Blood vessel density (mean ± SD) of immunostaining with CD31 of groups A–D at different times (n=6). ^a^P<0.05 vs. groups B–D; ^b^P<0.05 vs. 2 weeks; ^c^P<0.05 vs. groups C and D.

Fig. 4a shows the immunofluorescent stained sections of the implanted site at 2, 4 and 8 weeks after implantation. At 2 weeks, the grafts in rabbits from group A were filled by a few vessel lumina. No vascular endothelial cells were observed in groups C and D, although in group B there were few CD31-positive cells in the two ends of the graft. In rabbits from groups C and D, a few CD31-positive cells were found in the tibial and femoral ends. In group A, CD31-positive cells were observed in the core of graft at 4 weeks; however, vessel lumina were observed at the center of the graft in groups C and D at 8 weeks while CD31-positive cells in the grafts from group A were less than that at 4 weeks.

The data for microvessel density revealed that more significant neovascularization occurred in group A than in the other groups at every time point (Fig. 4b). Over time, the microvessel density increased gradually in groups B–D with significant statistical significance. Also, the microvessel density decreased in group A at 8 weeks, but there was no significant statistical significance (Fig. 4b).

## Discussion

In this study, we indicated that VEGF enhances revascularization of allografts after ACL reconstruction, which was similar with previous studies (11). Additionally, we found that application of SH to VEGF was useful by acting as a drug-release system of VEGF, leading to an acceleration in the process of graft remodeling.

Previous studies have shown that ligament healing is a complex and multistage process, which is controlled by a variety of factors. Growth factors are involved in the remodeling process and play an important role (12,13). VEGF is a potent direct angiogenic factor that stimulates endothelial cell migration and activation *in vitro* and *in vivo*. In addition, VEGF is a group of highly conservative secreted glycoproteins. Thus, in this study, human VEGF demonstrated beneficial biological effects in a rabbit model. Combined with a VEGF receptor, VEGF played an important role in inducing proliferation of endothelial cells in addition to promoting endothelial cell migration and survival. The angiogenic activation of endothelial cells probably plays a role in promoting and regulating other biological events, such as inflammation, fibroblast proliferation and extracellular matrix synthesis. We found that exogenous VEGF improved the early revascularization after ACL reconstruction using a B-PT-B allograft. Simultaneously, the peak blood vessel formation in group A occurred at 4 weeks. However, it declined at 8 weeks. The two reasons responsible for the aforementioned decline may be the following: i) the biological effect of one-time exogenous VEGF disappeared or ii) the body underwent its own adjustment; a deficiency in blood vessels in the normal ACL caused inevitable degradation.

However, the key for solving this problem is the short half-life of VEGF *in vivo*. We chose SH in this experiment for four reasons. Firstly, it has been widely used in ophthalmic surgery; no serious side-effects have been noted, it is well-tolerated, non-immunogenic and usually it does not cause any inflammatory reactions. Secondly, SH is a glycosaminoglycan and plays a protective, shock-absorbing and structure-stabilizing role in connective tissue. Thirdly, SH is biodegradable. After a certain period of time, the gel decomposes to biocompatible materials or is absorbed into the surrounding tissues, disappearing from the site of administration. The gel becomes progressively less viscous *in vitro* and begins to dissolve during a period of 1–2 weeks. Fourthly, SH could be used as a controlled and localized delivery system in a variety of conditions including osteoarthritic pain, basal cell carcinoma and actinic keratosis (8). It can be combined with certain polymers by covalent bonding and wraps the drug *in vivo* to form a slow-release system. Matsumoto *et al* (9) found that morphine intestinal suppositories had slow-release characteristics after adding 3% of SH. Surendrakumar *et al* (14) reported that recombinant human insulin combined with SH could make average residence time and half-life longer than pure insulin. In our study, we found that the SH system displayed diffusion controlled limitation of VEGF release. A sustained release was achieved over several hours. In addition, VEGF with SH obviously promoted revascularization, more effectively than pure VEGF (P<0.01). There was no significant difference between groups C and D (P>0.05). This revealed that SH itself could not promote revascularization, but it could prolong the average residence time and enhance the effect of VEGF.

In addition, we found that the ultimate failure load of the allograft soaked in VEGF solution was significantly lower than that of the allograft soaked in SH or PBS solution 2 weeks after ACL reconstruction. Meanwhile, the ultimate failure load of groups A and B became significantly higher than that of the other groups at 4 and 8 weeks. Therefore, we determined that the biomechanical characteristics of the graft decreased at an early phase and then increased apparently later by using exogenous VEGF. We considered that there may be two reasons for this phenomenon. Firstly, a number of newly formed vessels and infiltrative cells, which were induced by VEGF administration, decreased the density of the graft and enhanced the deterioration of the mechanical properties of the grafted tendon. Secondly, it has been reported that VEGF promotes matrix metalloproteinases (MMPs) which may directly digest the matrix of the graft (15,16).

Moreover, it is important to reduce the effect of the allograft itself in the process of revascularization. It is known that B-PT-B allograft transplantation may cause inflammatory rejection responses that may influence revascularization, and it also increases the risks of disease transmission. Freezing at −80°C can damage the fiber cells of the graft that are the main resource of normal MHC antigens. Consequently, graft antigencity is reduced (17). Pinkowski *et al* (18,19), Arnoczky *et al* (1) and Shino *et al* (13) did not detect any immune rejection response after transplantion of the allograft pretreated by deep freezing at −80°C. As for the risks of disease transmission, γ irradiation is now widely used as a safe and effective secondary sterilization technique (20). In this study, the B-PT-B allografts we used were pretreated by γ irradiation and stored at −80°C for 3 months. We found that there was no refection response phenomenon after reconstruction using B-PT-B allograft in all 45 rabbits.

In conclusion, our study suggests that SH can be used as an excellent carrier of VEGF by enhancing the effect of VEGF on revascularization and the biomechanical properties of grafts. This may be a useful method for improving the graft healing quality after ACL reconstruction.
